# Funtional Dyspepsia, So-called

**Published:** 1894-01

**Authors:** R. C. M. Page

**Affiliations:** Professor of General Medicine and Diseases of the Chest in the New York Polyclinic


### Selections and Abstracts.

### FUNCTIONAL DYSPEPSIA, SO-CALLED.

BY R. C. M. PAGE, M. D.

**Professor of General Medicine and Diseases of the Chest in the New York Polyclinic.**

There are two principal varieties of the so-called functional dyspepsia—irritative and atonic. In both the real pathological element we have to deal with is often a subacute or chronic gastro-intestinal catarrh. In the former class we find patients who are not infrequently robust and addicted to drink, gluttonous habits, or the eating of highly seasoned food and the like. In the latter are found those who suffer with a general lowering of the vital powers.

But in either case there is a deficiency in quantity and quality of gastric juice, and an abnormal increase of alkaline mucus which gives rise to fermentation rather than digestion. The stomach becomes distended with gas, so that peristalsis is impaired and there is obstruction to absorption of the stomach contents. Moreover the food becomes so enveloped in this alkaline mucus that the gastric juice fails to penetrate it and reach the food. Hence in such cases we would naturally expect to find a tongue more or less furred, bad taste in the mouth, and that the patient complained of a sense of fullness and distress at the epigastrium after meals. There are also sour stomach and sometimes heart-burn or cardialgia. This sensation not duerto excess of acid of the gastric juice—for that is really is diminished—but to lactic and butyric acids formed from the starcy foods instead of the latter being converted into glucose. Occasionally nausea and vomiting occur, notably in the morning, as in the vomiting of drunkards—or water brash.

Dyspnoea is not uncommon, and even paroxysms of peptic asthma may occur. Besides dyspnoea there are not infrequently palpitation and irregular action of the heart, with intermittent pulse. In fact, dyspepsia is one of the most frequent causes of intermittent pulse. These symptoms—dyspnoea and irregular cardiac action—are due partly to pressure from the distended stomach, and partly to reflex action along the pneumo-gastric and phrenic nerves. The patient often complains of vertigo. The bowels are alternately loose or constipated.

In the treatment of these cases, removing the cause and regulating the diet are of prime importance. As for removing the cause, this is often impossible in case the dyspepsia be associated with organic disease such as carcinoma, phthisis, and diseases of the liver, especially those that cause obstruction to the portal circulation. Mitral obstruction or regurgitation gradually brings about this condition. In the former case the blood is prevented from leaving the lungs, in the latter it is regurgitated back on the pulmonary circulation. In either case a strain is put upon the right ventricle and finally the liver itself, together with the whole gastro-intestinal tract, becomes conjested, giving rise to chronic catarrh of the stomach and bowels. General emphysema brings about the same result. Here we have obstruction to the pulmonary circulation, due to loss of capillary area in the lungs from ovordistension of the air-cells. In all such cases it is impossible, as a rule, to remove the cause.

But in irritable dyspepsia, dependent on alcoholism for instance, the cause can, and should be removed. In the same way the opium habit must be dropped. It may be asked how can opium, which generally causes the atonic form, give rise to irritative dyspepsia? Evidently by paralyzing the muscular coat of the stomach, thus allowing food to remain too long in that organ and so to cause irritation of the mucus membrane. Gluttonous habits should be modified and the use of improper and highly seasoned food, or food that is improperly cooked, be discontinued. So much for removing the causes of irritative dyspepsia. When we come to the antonic form, we find that here, too, the cause can often be ascertained and modified if not removed entirely. As such may be mentioned depressing emotions from loss of loved ones, financial ruin, the so-called disappointment in love, political aspirations and the like. As anything that lowers the vitality is likely to give rise to atonic dyspepsia; it should always be looked for and treated, as the dyspepsia is only a symptom of the real disease. Menorrhagia, excessive sexual indulgence, overwork with insomnia, as well as lack of sufficient and wholesome occupation, all tend to lower vitality and should be considered.

Space is wanting to refer to regulating the diet in full. In general terms, however, it may be said that in any case, meals should be regular, and the food eaten with deliberation instead of being swallowed hurriedly, especially when mentally worried about some scheme or other. In atonic dyspepsia, as a rule, tonic treatment is indicated and the patient has to build up. But an irritative dyspepsia the very opposite course is often to be pursued, care being taken especially to avoid starchy and fried foods and fats, as well as alcoholics.

Besides removing the cause and regulating the diet, many remedies have been suggested and tried by different practitioners from time to time. Of these remedies I do not hesitate to recommend papoid as one of the best. It dissolves the abnormal mucus secretions, thus removing a prime cause of fermentation besides stripping the food of its mucous envelop and thus exposing it to the action of the gastric juice. In addition to its direct digestive action on the stomach's contents, it also seems to have a stimulating effect upon the gastric mucus membrane. Its therapy is not materially interfered with by any of the drugs usually given internally, and it is equally efficacious in acid, alkaline, or neutral media. That it is not destroyed in the stomach like animal pepsin, is proved by the fact that even in ordinary doses a trace of it may be found in the stools, thus showing that the whole gastro-intestinal tract has received the benefit of its action. In cases where diminished peristalsis is marked, accompanied by accumulations of gas in the stomach and bowels, it is well to add strychnia. The average dose of papoid is about one and a half to three grains ter die after meals, and one of the most convenient methods for its administration is in tablet form.

Along with papoid other remedies may be used. One of the best, in many cases, is the rhubarb and soda mixture with tincture of nux vomica, or if the case is one due to abuse of alcohol (rum stomach) tincture of capsicum should be added. Where palpitation and irregular heart's action are present the tincture of digitalis may be added to the same mixture (Prescription—Tinct. digitalis 1 dr., pulv. rhei. sodii bicarb. of each 2 scr., aquae q. s. ad. f. 2 oz., m. sig. 1 dr. ter die). Washing out the stomach by means of the stomach tube was once much in vogue, but now it is used chiefly in those cases where dilatation is marked as in obstruction to the pylorus from cancer or other cause.

Besides actual treatment, patients who can afford it, often improve, or are perfectly cured, by taking a course at some watering place, care being taken to make a proper selection, which can only be done by advice of some physician who is intelligent on such subjects. The visiting of watering-places at random and the use of the waters without proper care and instruction is undoubtedly more productive of harm than good.

Before leaving this subject a word of warning to physicians and their patients. Alcoholics and opiates may be needed at times in the treatment of these cases, but beware of their repeated and too free use. It is by these small beginnings, and in such apparently innocent schools, that the drunkard and opium fiend are educated. The fatal mistake should not be made of bringing about some habit worse than the original disease, and more difficult to cure.—*The New York Polyclinic.*

